# Exploring attitudes toward fertility and childbearing among married women in Kabul, Afghanistan: a qualitative study

**DOI:** 10.1186/s12978-025-02231-7

**Published:** 2025-12-10

**Authors:** Ziba Mazari, Seyedeh Tahereh Mirmolaei, Masud Yunesian, Shirin Shahbazi Sighaldeh, Sadaf Sultani, Halima Baha, Sodaba Mohammadzai

**Affiliations:** 1 Department of Reproductive Health and Midwifery, School of Nursing & Midwifery, Tehran University of Medical Sciences, Tehran, Iran; 2 Department of Enhanced Midwifery Skills, School of Midwifery, Kabul Medical University, Kabul, Afghanistan; 3 School of Public Health, Tehran University of Medical Sciences, Tehran, Iran; 4 Nursing and Midwifery Care Research Center, Tehran University of Medical Sciences, Tehran, Iran; 5 Department of Normal Midwifery Care, School of Midwifery, Kabul Medical University, Kabul, Afghanistan; 6 School of Midwifery, Alberoni Institute of Health Science, Kabul, Afghanistan

**Keywords:** High fertility, Fertility attitudes, Childbearing motivations, Reproductive agency, Birth spacing, Family planning, Afghan women in Kabul

## Abstract

Afghanistan faces persistently high maternal mortality, high fertility, and low use of modern contraceptives—trends at risk of worsening under current restrictions on women’s mobility, education, and access to health services. Although family planning is a cost-effective strategy in high-fertility, low-resource settings, generating demand has long remained a challenge in Afghanistan. While socio-cultural barriers to family planning are well documented, limited evidence captures how women themselves interpret and negotiate fertility and childbearing within these constraints. This study explored married women’s attitudes toward fertility and childbearing in Kabul to inform locally appropriate approaches to strengthening reproductive well-being.

**Methods **In 2024, in-depth semi-structured interviews were conducted with 23 married women aged 20–43 years (mean = 32.1) in Kabul, Afghanistan, purposively selected for demographic diversity. Interviews were audio-recorded when possible or otherwise documented in detailed notes, and analyzed concurrently in MAXQDA 2024, with data collection continued until no new codes or insights emerged across three successive interviews.

**Results **Five major categories were identified: (1) socio-cultural norms and expectations, (2) religious and ethical perspectives, (3) economic and functional dimensions of childbearing, (4) health and well-being considerations, and (5) emotional and psychological motivations. Across interviews, pronatalist norms and expectations for early and repeated childbearing remained dominant and were reinforced by misconceptions about contraception. Nevertheless, some women emphasized maternal health, child well-being, and more balanced decision-making within families—reflecting a diversity of reproductive perspectives within the prevailing social context.

**Conclusions** Women’s fertility attitudes reflected the coexistence of enduring pronatalist expectations with value-oriented considerations emphasizing maternal and child well-being. These perspectives illustrate nuanced forms of reproductive reasoning that may inform culturally responsive approaches to reproductive health promotion. Future research should examine how such orientations vary across Afghan settings. Within the current social constraints, discreet and context-appropriate counseling integrated into existing maternal and child health services could help support informed fertility decisions and contribute to safer maternal outcomes.

## Introduction

 Afghanistan has one of the highest maternal mortality ratios (MMR) in the world, with 638 deaths per 100,000 live births [1]. Persistently high fertility—combined with limited healthcare capacity [2]—continues to contribute to poor maternal and child health outcomes [3–5]. Although family planning is widely recognized as a cost-effective strategy for reducing maternal mortality, particularly in high-fertility, low-resource contexts [6–8], generating sustained demand for modern methods has long been a challenge in Afghanistan due to persistent socio-cultural barriers and limited community engagement [9, 10]. Such barriers, rooted in pronatalist norms and values, gendered decision-making, and limited communication about contraception, have been reported across different regions of Afghanistan [2, 11–15]. Recent restrictions on women’s mobility, coupled with declining donor support for the health sector and fewer educational and employment opportunities, have further constrained women’s access to reproductive health information and services, widening the gap between reproductive needs and the ability to act upon them [9, 16, 17].

Beyond the healthcare sector, rapid population growth—estimated at 2.3% annually [18]—adds pressure to an already fragile system, which is heavily reliant on external aid. These strains have intensified since 2021 reductions in international support and the downsizing of reproductive, maternal, and child health programs. Following the 2021 political transition, most development aid was suspended because the new authorities were not internationally recognized. Donors cited concerns over governance, accountability, and restrictions on women’s rights [19–21]. Consequently, large development programs were halted, leaving only limited humanitarian assistance through UN agencies and NGOs.

While the previous administration identified family planning awareness and demand generation as national priorities [15], the current de facto government has not articulated a clear reproductive health policy. Media reports have described temporary and localized restrictions on contraceptive distribution and women’s mobility [16, 22]. Nevertheless, recent assessments indicate that contraceptive services remain available in most urban and many NGO-supported rural facilities, although outreach and supply regularity have weakened [9, 17], especially in remote areas where mahram requirements and the shortage of female health providers further reduce women’s access to available services. Alongside these challenges, some community- and health-based initiatives led by national and international NGOs continue to provide limited reproductive health and livelihood services for women through local clinics and outreach activities [23–25]. This continued, though reduced, availability offers a narrow yet vital opportunity to reinforce awareness and sustain health-protective practices through existing service and support channels.

Existing research on fertility and family planning in Afghanistan is mostly quantitative, examining factors such as women’s education, household wealth, media exposure, parity, and participation in household decision-making. These studies consistently show high fertility with only modest variation by socio-economic status; even among educated, wealthier, or urban women, fertility remains well above replacement levels [13, 26–28]. Successive national surveys—including DHS 2015, AfHS 2018, UNFPA 2022, and MICS 2022–23—report total fertility rates around 5.3–5.4 and modern contraceptive prevalence of 18–20%, with only modest urban–rural differences (TFR ≈ 4.3–4.8 in urban areas and 5.4–5.8 in rural areas, mCPR ≈ 17–23%)—confirming persistently high fertility and low contraceptive use nationwide [9, 29–31].

The need for further qualitative work to unpack the sociocultural and motivational dimensions of fertility has therefore been widely emphasized by both researchers and program stakeholders [9, 10, 26, 32]. Early programmatic analyses identified multiple social and cultural barriers to family planning—including son preference, limited male involvement, and misconceptions about side effects—based mainly on interviews with program managers and health workers in several provinces, including Kabul [33]. A 2009 qualitative study conducted in Kabul among postpartum couples found some awareness of the benefits of birth spacing among women but also demonstrated that male dominance and pronatalist expectations remained pervasive [34]. Later qualitative studies from various Afghan provinces—several of which also included participants from Kabul—mainly relied on focus group discussions and emphasized stakeholder perspectives rather than women’s own narratives. These studies consistently highlighted limited female autonomy, the strong influence of husbands and extended families, misconceptions about contraceptive side effects, and moral or religious concerns surrounding family planning [11, 12]. More recent data from the UNFPA (2022) Behavioural Study offer valuable yet limited insight into women’s reproductive reasoning in the post-transition context. While primarily quantitative, its small qualitative component confirmed the persistence of male disapproval, family pressure, and misconceptions, alongside emerging health-protective orientations among some younger and urban women who viewed birth spacing as beneficial for maternal recovery and children’s well-being [9].

However, despite growing documentation of service-related and behavioral barriers, most existing qualitative studies have relied primarily on focus group discussions and stakeholder accounts rather than on women’s own narratives. Consequently, there remains a lack of in-depth evidence on how Afghan women interpret and navigate fertility and childbearing within the socio-cultural and moral systems that shape their lives. Understanding women’s lived experiences and reasoning is essential to inform culturally grounded and feasible reproductive health strategies that promote health-oriented fertility choices. Accordingly, this study aimed to explore married women’s attitudes toward fertility and childbearing in Kabul, Afghanistan, by examining how they perceive and respond to societal expectations, economic conditions, and cultural norms, alongside their personal needs in shaping values and choices related to fertility.

## Materials and methods

This qualitative study was conducted in Kabul, Afghanistan, in 2024, utilizing in-depth, semi-structured interviews as the primary method of data collection. The study focused on married Afghan women of reproductive age, selected to provide diverse insights into individual attitudes toward fertility and childbearing.

### Sampling and setting

A non-probability purposive sampling approach was used to ensure maximum variation within the target population. Participants were primarily recruited from among women accompanying patients at three public hospitals in Kabul: Rabia Balkhi, Isteqlal, and Shahr-e-Ara. These referral hospitals offered access to a more demographically diverse population. Eligible participants were defined as married women aged 15 to 45 years who were not pregnant, had no clinically confirmed infertility or health condition that prevented meaningful participation, were mentally capable of engaging in an interview, spoke Dari, and consented to join the study.

To enhance sample variation, participants were purposively selected based on key demographic factors, including age, education, occupation, economic status, place of residence, and ethnicity, with priority given to individuals who could contribute to this diversity. A referral-based recruitment approach was also used, with colleagues, friends, and acquaintances suggesting eligible participants. Of the 28 women approached, 5 declined to participate for personal reasons —mainly due to limited time availability, family disapproval, or insufficient rapport or trust to feel comfortable participating —resulting in a final sample of 23 participants (17 recruited through hospitals and 6 through community contacts). No participants withdrew once their interviews began.

### Consent and confidentiality

Informed consent was obtained by briefly explaining the study’s purpose, as well as its potential benefits—including the intended contribution to understanding women’s perspectives on fertility and to future community efforts aimed at supporting women’s well-being—along with key features of participation, such as audio recording and confidentiality (e.g., anonymity and deletion of recordings), and the voluntary nature of the involvement with the option to withdraw at any time. To ensure confidentiality and participant safety, each participant was assigned a unique study code. Providing a real name was optional; participants could use a pseudonym if preferred. Personal contact information was collected only when follow-up communication was needed and with the participant’s consent, while the researcher’s contact number was provided to all participants for any questions or concerns. These measures were implemented to minimize any perceived risk or hesitation about participating.

Two trained research assistants facilitated the consent process—especially for audio recording—by clearly explaining study procedures until participants felt comfortable. The research team worked collaboratively to create a respectful and supportive environment for obtaining fully informed consent. Verbal consent was used because many participants were illiterate or preferred not to sign formal documents; it was documented either as a consent statement recorded at the start of the interview or as a witness signature by a research assistant indicating that oral consent had been provided. Due to cultural sensitivities, three participants declined audio recording; in these cases, detailed manual note-taking was conducted.

Interviews were held at locations mutually agreed upon with participants, most often in private rooms within hospitals, and when this was not feasible, in another hospital, or any nearby private setting, to ensure privacy and minimal interruptions. No third party was present during any interview. There was no pre-existing relationship between the interviewer and participants. To protect confidentiality and maintain feasible participation, no routine follow-up was conducted.

### Data collection

Data were collected through 23 semi-structured, face-to-face interviews conducted between March and October 2024. The interview time ranged from 35 to 90 min (about 22 h in total). The interviews were conducted by the first author (ZM), an Afghan female researcher with a Master’s degree in midwifery and currently pursuing a PhD in reproductive health, trained in qualitative methods. As a midwife with professional experience in the same context, she shared the participants’ gender, language, and cultural background, which made her familiar with cultural sensitivities and adept at effective communication. Each interview began with rapport-building through culturally appropriate greetings and brief friendly exchanges to create a comfortable environment for discussing personal and semi-private topics. Before the main interview, a short demographic questionnaire was administered, with the researcher reading the questions aloud and recording participants’ responses, to collect information on age, education, occupation, and reproductive history (e.g., number of pregnancies, living children, contraceptive use).

The main qualitative interviews were conducted using a semi-structured interview guide (see Table 1) to explore women’s attitudes toward fertility and childbearing. The interview guide was developed by two researchers (ZM and SSS) based on the study objectives and a review of relevant Afghan and international literature. Elements drawn from the Value of Children theory and the Theory of Planned Behavior informed some questions, though the guide was not restricted to these frameworks. The questions were designed to capture women’s perceptions, emotions, beliefs, and choices regarding fertility and its influencing factors. The guide was part of the broader study protocol, which was reviewed by a team of experts with backgrounds in reproductive health and/or epidemiology. Minor modifications were made after the first few interviews to improve clarity, neutrality, and alignment with the study aims. The original interview guide was developed in Dari, using language close to everyday spoken expressions, and the version presented in the manuscript is its English translation. According to the guide, the exploration began with two contextualized opening questions — “Do you want to have a/another child in the future?” and “How many children would you like to have?” — followed by other open-ended questions. Probing techniques were applied to deepen participants’ reflections and clarify meanings. During the interviews, the researcher continuously checked the participant’s comfort and willingness to proceed, maintaining rapport and ensuring a natural flow of conversation through empathetic listening and reflective prompts. She remained reflexively aware of her role during the interviews and took care not to influence participants’ responses or lead their accounts.

**Table 1:** Interview guide

1. Do you want to have a/another child in the future? Why or why not? 2. How many children would you like to have, and what gender composition do you prefer? Do you have any preference for sons or daughters? Why? 3. In your opinion, what is the ideal number of children for a family? At what age do you think women should marry, and when do you think they should ideally have their first child? What do you believe is the appropriate spacing between births, and why? 4. Have you ever done anything to prevent pregnancy? What has been your experience or feelings about using family planning methods? 5. Have you and your husband ever discussed how many children to have or whether to use family planning methods? How did those conversations go? How much say do you feel you have in making these decisions? 6. How important is having children in your life? Why? 7. When you think about having children, what kinds of emotions or thoughts come to mind? 8. How do pregnancy, childbirth, and raising children affect a woman’s life? How has your own experience been? Has it influenced your decisions about having (more) children in the future? 9. Do you feel that having children brings more benefits or more challenges? Why? How do these affect your choices about having children? 10. How do you think the presence or number of children affects the family’s life? 11. What do you believe religion says about having children and about preventing pregnancy? How much do you personally follow those religious beliefs in your life? 12. How do people around you—like your family, friends, or neighbors—usually think about having children? Have they influenced your childbearing decisions?		

A research assistant transcribed the audio-recorded interviews soon after completion and took detailed notes during sessions where recording was not permitted. The first author reviewed all notes and transcripts, cross-checking them with audio files and participants’ statements to ensure completeness and faithfulness to participants’ narratives. At the end of each interview, a brief summary of key discussion points was shared with and confirmed by the participant as part of member checking, while transcripts of three interviews were later returned for additional validation as a procedural check to confirm that the note-taking and transcription process accurately reflected participants’ accounts, given that full transcript review by all participants was not feasible due to practical constraints. Each participant was interviewed once; no repeat interviews were conducted.

The credibility and dependability of the findings were further supported by detailed field and reflexivity notes maintained by the first author, complemented by peer debriefing within the research team and by the first and corresponding authors’ collaborative review of the research process. These activities informed iterative refinements to study procedures, including adjustments to the interview guide, consent processes, and approaches when audio recording was not permitted, as well as the types of participants needed to enrich diversity and to ensure interviews were conducted in a neutral and professionally appropriate manner. The study protocol was reviewed and approved by the Ethics Committee of Tehran University of Medical Sciences (Code: IR.TUMS.REC.1402.239).

### Analysis

The study employed conventional content analysis following the approach described by Zhang and Wildemuth [35]. Data were managed using MAXQDA-24. Interviews were transcribed in Dari, while coding and content analysis were performed in English.

The first author, who conducted all interviews and was closely familiar with the study context, immersed herself in the transcripts through repeated reading and led the primary coding and interpretation. Her close and sustained engagement with the data, supported by detailed field and reflexive notes, contributed to contextual sensitivity and analytical coherence while she remained attentive to how her positionality might influence interpretation.

Interviews were conducted, transcribed, and coded in a sequential, iterative cycle, such that each interview entered analysis before the next was undertaken. During the coding process, meaning units—segments relevant to the study’s objectives—were identified and labeled with descriptive codes that captured their core ideas. The corresponding author independently coded five transcripts, and any discrepancies were resolved through discussion until consensus was reached; this process helped maintain coding consistency across the dataset.

The initial coding scheme was developed from several transcripts, iteratively refined, and gradually expanded as the analysis progressed. Team discussions were held periodically to review code naming and grouping and to resolve interpretive differences. Saturation was monitored continuously by comparing codes from each interview with those of preceding interviews. Data collection continued until no new codes or insights emerged across three successive interviews, at which point data saturation was considered achieved.

The resulting coding tree was organized into a three-level structure—categories, subcategories, and codes—to systematically present the data and clarify relationships among analytic units. The research team jointly reviewed the final analytic framework and the resulting interpretations to enhance credibility and analytical rigor. Participant feedback on preliminary themes or summaries was not obtained due to practical constraints. Although the article reports on nearly all identified codes, a few less prominent codes are presented in detail in the related dissertation due to space limitations.

No theoretical framework was applied during coding; categories were derived inductively from participants’ data through a low-inference, data-driven analytic approach, while existing literature and theoretical perspectives were engaged only in the Discussion to contextualize the findings, interpret differences and variations across participants’ perspectives, compare them with prior work, clarify the relevance of emerging insights and theoretical perspective, and identify practical implications for strengthening communication and support within the current reproductive-health context, without influencing coding decisions or shaping category development.

## Results

Findings are based on 23 in-depth interviews with married Afghan women aged 20–43 years (mean: 32.1). All resided in Kabul province, including 20 from urban areas and three from rural districts. Twelve participants were illiterate, four had higher education, and seven had primary or secondary schooling. Most were unemployed housewives; four were formally employed, and four engaged in income-generating home-based work. Nineteen participants identified as Sunni and four as Shia, representing diverse ethnic backgrounds. The demographic and reproductive characteristics of participants are presented in Table 2.

**Table 2:** Study participants' demographic profile

Participant’s ID Number	Age (years)	Education	Husband’s Education	Occupation	Husband’s Occupation	Province of Origin	Religion	Ethnicity	Number of Children	
P.1	36	Illiterate	Illiterate	Housekeeper	Unemployed	Maidan Wardak	Shia	Turkmen	2	
P.2	29	Associate Degree	High School	Teacher	Car Painter	Kabul	Sunni	Tajik	1	
P.3	33	Illiterate	Illiterate	Housewife	Government Office Cleaner	Parvan	Sunni	Tajik	7	
P.4	39	Illiterate	Illiterate	Housewife	General Laborer	Samangan	Sunni	Tajik	8	
P.5	40	Illiterate	Illiterate	Home-based Tailor	Street Vendor	Panjshir	Sunni	Tajik	7	
P.6	36	Illiterate	Illiterate	Home-based Baker	Street Vendor	Kapisa	Sunni	Pashtun	5	
P.7	20	Primary School	Middle School	Housewife	Street Vendor	Kabul	Sunni	Tajik	1	
P.8	25	Middle School	Illiterate	Housewife	Carpenter	Maidan Wardak	Sunni	Tajik	3	
P.9	36	Illiterate	Illiterate	Home-based Dairy Farmer	Home-based Dairy Farmer	Kabul	Sunni	Tajik	5	
P.10	31	Illiterate	Illiterate	Housewife	Chef	Kabul	Sunni	Tajik	6	
P.11	26	Illiterate	Illiterate	Housewife	Shopkeeper	Kabul	Sunni	Pashtun	4	
P.12	39	High School	Bachelor’s Degree	Housewife	Herbal Medicine Seller	Kabul	Sunni	Tajik	6	
P.13	32	Middle School	Middle School	Housewife	Shopkeeper	Maidan Wardak	Sunni	Turkmen	5	
P.14	33	Illiterate	Illiterate	Housewife	Construction Worker	Kapisa	Sunni	Pashtun	5	
P.15	41	Primary School	Middle School	Housewife	Freelancer	Ghazni	Shia	Hazara	5	
P.16	22	Illiterate	Master’s Degree	Housewife	Car Mechanic	Bamiyan	Sunni	Tajik	2	
P.17	38	Primary School	Bachelor’s Degree	Housewife	Retired (Security Officer)	Kunduz	Sunni	Pashtun	4	
P.18	36	Bachelor’s Degree	Bachelor’s Degree	Teacher	Taxi Driver	Ghazni	Shia	Hazara	4	
P.19	30	Bachelor’s Degree	Bachelor’s Degree	Laboratory Technician	Owner of Veterinary Pharmacy	Maidan Wardak	Shia	Hazara	3	
P.20	27	Illiterate	Middle School	Housewife	Auto Mechanic	Kabul	Sunni	Tajik	3	
P.21	43	High School	Bachelor’s Degree	Home-based Tailor	Retired (Military Officer)	Kabul	Sunni	Pashtun	6	
P.22	22	Illiterate	Bachelor’s Degree	Housewife	Prosecutor	Jalalabad	Sunni	Pashtun	3	
P.23	24	Primary School	Primary School	Housewife	Goldsmith	Ghor	Shia	Hazara	4	

Analysis yielded a comprehensive set of codes capturing participants’ beliefs, emotions, and preferences regarding various dimensions of childbearing and fertility. The data were organized into five main categories and fifteen subcategories, reflecting socio-cultural, religious, economic, health-related, and emotional dimensions of women’s fertility-related views (see Table 3). Each category is explored below through participants’ narratives. All quotations in this section were translated into English by the first author, who is fluent in both Dari and English, to ensure accuracy and preservation of meaning.

**Table 3:** Categories and subcategories

Main Category		Subcategory	Codes	
1. Perceived Socio-cultural Norms and Expectations around Childbearing		1.1. Social norms and pressures for large families	Desire for many children • Men prefer more children • Pressure from in-laws and community • Comparing family sizes across generations	
		1.2. Expectations for early first pregnancy	Immediate pregnancy after marriage • Criticism for delays • Linked to marital stability • Avoiding contraception before first birth	
		1.3. Stigma of infertility and sonlessness	Infertility shame • Social stigma • Pressure to have sons • Sons valued for lineage	
		1.4. Emerging attitudes toward family size	Preference for fewer children • Prioritizing children’s well-being and development • Manageable lifestyle	
		1.5. Conditional agency in fertility decisions	Negotiating number of children • Secret contraception use • Joint decision-making	
		1.6. Persistent son preference	Initial idealized gender balance • Spousal or family strong insistence on one or two sons • Larger families to ensure sons	
2. Religious Beliefs and Ethical Perspectives		2.1. Divergent religious views on family planning	Contraception sinful • Contraception permissible • Conditional acceptance • For spacing rather than limiting • Uncertainty about religious rulings	
		2.2. Religious motivations for childbearing	Children as God’s blessings • God as provider • Raising children as worship	
		2.3. Balancing faith and practical needs	Accepting sin due to financial hardship • Not fulfilling children’s needs: real sin • Balancing with maternal health	
3. Economic and Functional Dimensions of Childbearing		3.1. Financial burden of raising children	High child-rearing costs • Effects of unemployment • Adjusting family size to resources • Impact of rising living standards	
		3.2. Functional value of children	Functional contributions • Care for parents in old age • Sons as long-term security • Changing daughters’ roles • Shift toward individualism • Child-centered values	
4. Health and Well-being Considerations		4.1. Physical and emotional burden of motherhood	Physical exhaustion • Emotional strain • Heavy household workloads • Family conflicts • Spousal & family support • Neglected maternal health • Economic impact • Favorable conditions ease burden • Resilience of older mothers • Younger women’s body image concerns	
		4.2. Birth timing and spacing preferences	Preferred 2–3 year gap • Three-year gap after C-section • Longer spacing for child independence • Positive views on early childbearing • Shorter spacing due to maternal age	
		4.3. Concerns about modern contraceptives	Fear of side effects • Misconceptions • Discontinuation due to problems • Preference for natural methods	
5. Emotional and Psychological Motivations of Childbearing		5.1. Joy and companionship	Children as happiness • Filling emotional gaps • Children as lifelong companions • Balanced joy with child-rearing responsibilities	
		5.2. Fulfillment and marital stability	Motherhood as identity • Children as marriage bond	

### Perceived socio-cultural norms and expectations around childbearing

Findings indicate that family and community expectations play a major role in shaping women’s fertility attitudes and reproductive choices.

#### Social norms and pressures for large families

While most women in this study expressed a desire to limit childbearing, ‘limiting’ often meant having four to six children. Several participants emphasized that having many children was still regarded as the norm in their communities, as one woman explained, *“Others have six*,* seven*,* or eight children—no one has fewer than six. Younger couples also have three*,* four*,* or five”* (P.15). Another participant compared this to the past, noting, *“Before*,* families used to have ten or eleven kids*,* but now most prefer four or five”* (P.7). These comments suggest that while participants observed a gradual shift toward smaller families, having several children was still perceived as a prevailing social norm within their communities.

Some participants attributed the preference for a large family to their husbands’ expectations:

*“My husband says I should have many children. He says*,* for example*,* ‘Have ten.’ But I can’t — I just can’t have ten children.”* (P.16).

Others described this expectation as widely shared among men in their communities:

*“Men usually prefer having more children. They say*,* ‘Just as a king never gets tired of ruling*,* a man never gets tired of having kids.’”* (P.5).

Several women reported pressure from husbands and in-laws to have more children, as one woman shared, *“My husband and his family insisted*,* saying they wouldn’t settle for just four children. I myself didn’t want to have more than four but because of them I ended up having six”* (P.12). The same participant also described broader social pressure and public questioning when she delayed having another child: *“People questioned me because my daughter was already six years old*,* and everyone kept saying I should have another baby”* (P.12).

#### Expectations for early first pregnancy

Most participants reported experiencing their first pregnancy soon after marriage. Several described strong social expectations for immediate pregnancy—locally referred to as ‘haml-e sar takht’ (literally, ‘pregnancy on the wedding bed’). Some participants described experiencing criticism or pressure from in-laws when pregnancy was delayed:

*“When I first got married and hadn’t had any children yet*,* my mother-in-law and sometimes my father-in-law would say*,* ‘If you don’t have children*,* I’ll find another wife for my son.’ “(*P.3).

These narratives indicate that early pregnancy is widely regarded as evidence of fertility and a source of marital stability and family approval. Some participants also mentioned a belief that using contraception before the first childbirth could increase the risk of infertility:

*“Mothers often worry that if their daughters use contraceptives right after marriage*,* they might not be able to get pregnant later*,* so they tell them not to.”* (P.15).

#### Stigma of infertility and sonlessness

Participants frequently emphasized that infertility is a deeply painful experience for women in their culture, often leading to marital instability and social ostracism:

*“Being childless is very difficult. People say ‘she’s not a real woman*,* that she’s like a stone*,* and her womb is barren’. Her husband may marry again.”* (P.22).

In addition, according to participants, the preference for sons also created persistent psychological pressure, as women with only daughters were often subject to subtle blame or devaluation within families and communities. The cultural emphasis on sons is closely tied to preserving the family lineage:

*“My sister had six daughters and no sons*,* and her husband was really worried about it. The family also talked about it a lot*,* saying things like*,* ‘His light will go out*,*’ meaning he had no sons to carry on his name*,* but after she gave birth to a son*,* people stopped talking. Now*,* she has two sons.”* (P.1).

#### Emerging attitudes toward family size

While large families remain a valued social norm, participants’ narratives suggest gradual shifts in fertility attitudes, illustrating changing lifestyle aspirations. As one woman noted, younger couples increasingly favor smaller families for a more manageable life:

*“They say ‘Less harvest*,* less loss; more harvest*,* more loss.’ By ‘loss*,*’ they mean the difficulties and burdens of raising children.”* (P.3).

Some participants also noted that their husbands preferred fewer children and limiting fertility, indicating a subtle shift toward valuing quality over quantity:

*“My husband says this is enough*,* so we can focus on their education and well-being. He believes having more children only creates problems*,* more conflicts and greater life difficulties.*” (P.17).

#### Conditional agency in fertility decisions

Participants’ accounts revealed a subtle interplay between personal preferences and familial expectations. While many women accepted a more deferred and accommodating role in decision-making — *“My husband wants more children*,* although I don’t agree*,* I say*,* ‘Alright*,* whatever makes you happy’” (P.9) —* some younger and more educated women shared experiences of negotiating their reproductive choices within family boundaries.

A 27-year-old participant recounted a discussion with her husband: *“Sometimes he says*,* ‘Let’s have two more children.’ But I tell him*,* ‘No*,* just one more*,*’ then he says*,* ‘Alright*,* that’s fine’”* (P.20). In another case, a 29-year-old woman with higher education and employment reflected a more differentiated dynamic, where specific aspects of reproductive decisions were openly negotiated: *“The final say on birth spacing and when to get pregnant is mine*,* but the number of kids is up to my husband and his family”* (P.2).

A home-based tailor explained how she learned about family planning methods through her clients:

*“I didn’t know about these family planning methods before. I learned about them from women who came to have clothes sewn. After I found out*,* I secretly took the tablets for a while. When I told my husband*,* he was fine with it.”* (P.5).

Finally, a small number of participants’ accounts indicate more communicative and joint forms of decision-making, where choices were openly discussed and mutually agreed upon, as a 20-year-old woman shared:

*“We talk about it together*,* and he also doesn’t like having many children. He says*,* ‘This one is enough; let’s stop here for the next ten years.’”* (P.7).

#### Persistent son preference as a driver of higher fertility

Several participants noted that, although they originally hoped for a balanced number of sons and daughters, spousal pressure and the desire to secure at least one or two sons led to larger family sizes:

*“I initially wanted four children*,* two sons and two daughters*,* but since God didn’t give me a son*,* I had to continue until He did. That’s why I didn’t start using contraception earlier—I wanted a son. My husband also wanted at least two sons. When God gave us two sons*,* we finally started using contraception after having eight children.”* (P.4).

### Religious beliefs and ethical perspectives

#### Divergent religious views on family planning

Fourteen participants held the view that preventing pregnancy is sinful and that childbearing should follow *‘Divine Will‘.* One participant explained:

*“I believe contraception is a sin. People say there was a woman who used birth control*,* and she had a dream where she lifted the lid of a pot and saw it filled with blood. She saw the Prophet Muhammad*,* who told her*,* ‘You have burned a servant of God with your own hands.’ I heard this story from the mosque cleric.”* (P.6).

Conversely, several participants explained that within Islamic teachings, contraception is considered permissible, while abortion is prohibited:

*“I believe our religion permits contraception*,* but only for spacing between children after having the first child. However*,* it should be used before conception. If you become pregnant and then terminate it*,* it is like being a murderer.”* (P.5).

#### Religious motivations for childbearing

In addition to opposing contraception, some participants referred to Islamic teachings—often voiced by family members—that discouraged limiting births:

*“My husband says*,* ‘Don’t stop having children. God grants children and provides for them*,* and the earth carries their burden. Don’t worry about financial problems—God is merciful.’”* (P.9).

Others described the spiritual rewards associated with raising children:

*“The mullah said caring for children is full of blessings. Even simple things—like giving your child water—count as worship… When a child prays*,* the reward also goes to their parents. Why would I be arrogant about a blessing God has given me?”* (P.6).

#### Balancing faith and practical needs

Despite holding anti-contraceptive beliefs, many participants nonetheless acknowledged the practical pressures that made family planning necessary. This often led to internal conflict, as expressed in their own words:

*“From my perspective*,* it’s a sin. I believe it shows arrogance. But what can we do? The economy is poor; we have no other choice.”* (P.16).

Others reframed the moral argument:

*“Some say contraception is a sin*,* some say it isn’t. But I think if I can’t provide for my children*,* that’s the real sin.”* (P.7).

### Economic and functional dimensions of childbearing

Beyond these religious and cultural influences, economic realities also emerged as a critical factor shaping fertility decisions.

#### Financial burden of raising children

Participants frequently reported that due to increasing costs of raising children—including food, healthcare, and especially education—they prefer fewer children. These concerns were exacerbated by rising unemployment and heightened aspirations to provide better education and living standards for children. One participant described the impact of reduced incomes:

*“Unemployment has risen*,* leading to lower incomes. No matter how many children one has*,* the reduced earnings make it difficult to provide for them.”* (P.3).

Another participant stressed the burden of education-related expenses:

*“I worry about my child’s education—public schools are affordable but lack quality education*,* while private schools offer better learning but come with high costs*,* which we must be able to afford.”* (P.2).

Nevertheless, a few participants with strong rural backgrounds held the traditional faith-based attitudes toward fertility:

*“We are not ungrateful for the children God grants us. When a child is born*,* we somehow manage. As they say*,* ‘We take one gulp and two bites’ (meaning*,* we make do with what little we have). Thank God*,* as they say*,* ‘Nan o piyaz bashad*,* ba peshaniy-e baz bashad’ (as long as there is bread and onions*,* and a smiling face*,* it is enough).”* (P.10).

#### Functional value of children

Although raising children was often seen as costly, many participants also believed that having children provides long-term economic benefits and future support:

*“In the early years*,* when a child is young*,* expenses are higher—covering basic needs*,* private schooling*,* and so on. However*,* in the future*,* having more children can be beneficial*,* as they will contribute financially and help support the family’s expenses.”* (P.2).

A common reason repeatedly mentioned by participants for having children was the expectation that they would later care for their parents: “*When we grow old and weak*,* these children are like fields and land—they will put a piece of bread in your mouth*” (P.3).

Sons’ perceived long-term contributions to family support appear to be a key factor underlying the previously noted preference for sons. However, no woman in this study explicitly expressed a personal preference for sons over daughters. Instead, they often echoed the perceived advantages of sons voiced within their communities or by their husbands: “*People like having sons; they say that a son will take care of us in the future*” (P.4); “*My husband wants a son because he believes a son is his father’s arm (aid). He says daughters belong to others and will leave*,* but a son stays with his family*” (P.9).

Participants also frequently explained that such beliefs are reinforced by prevailing norms that limit daughters’ ability to support their natal families after marriage:

*“If a child is good and responsible*,* their gender doesn’t matter. However*,* after a girl’s marriage*,* her in-laws may not allow her to care for her parents.”* (P.18).

A few women questioned the traditional preference for sons, citing changing economic realities and shifting gender roles. One woman noted that sons can no longer be relied upon to care for their parents, as *“everyone struggles to cover their own living costs”* (P.8). Another explained that *“there are families where daughters’ work supports household finances”* (P.2). This study also identified a more child-focused attitude among a small proportion of participants who prioritized their children’s lives and futures over the benefits the children might provide to their parents:

*“When my child grows up*,* they should study and pursue their dreams. Naturally*,* if they study*,* they will have an income. If they get an education*,* the mother will be happy. First*,* they should build their own life—after that*,* God is kind.”* (P.20).

### Health and well-being considerations

#### Physical and emotional burden of motherhood

Participants frequently described the combined demands of childbearing and continuous caregiving as both physically exhausting and emotionally draining. Many reported fatigue, anemia, and chronic pain caused by repeated pregnancies. As one woman explained: *“They say a mother is like a wall; with each child born*,* it is as if a brick is removed from that wall”* (P.2). Emotional exhaustion was also common: *“People don’t see the sleepless nights*,* the struggles*,* and the tiredness”* (P.4).

These burdens were often exacerbated by additional household duties and responsibilities within large, extended families common in Afghanistan. Living as a daughter-in-law with disproportionate workloads, one participant shared:

*“I have faced so many struggles—far too many. I couldn’t take care of my own health because our family was big; if I cooked*,* I couldn’t sweep*,* and if I swept*,* I couldn’t look after the kids. I’ve suffered mentally and have ended up in the hospital several times”* (P.17).

Family conflicts also restricted women’s ability to care for themselves and their children: *“A lot of controversies at home have affected me. We live in the same place with in-laws. My eldest daughter was malnourished*,* they were still sarcastic”* (P.14). In some cases, such tensions prompted women to limit childbearing: *“In our house*,* four brothers’ families live together*,* and constant fights among the children have exhausted me. That’s why I don’t want more children”* (P.11).

Despite these challenges, a few women highlighted supportive roles within families—for example, help from sisters-in-law or grown-up daughters with household chores (P.8, P.14). Similarly, some participants appreciated spousal support for their health and recovery: *“My husband helped me with taking care of the children”* (P.18); “*He says he wants my body to fully recover*,* so there should be a gap of at least five years before having another* child” (P.22). However, several participants expressed that their health needs were often neglected, as one woman shared experiencing pressure to have more children despite physical strain: *“Having too many children causes leg and back pain*,* but I have to keep having children because my husband insists”* (P.9).

Some women attributed the hardships of motherhood to poor economic and living conditions: *“Motherhood itself didn’t pressure me much*,* but financially it has been very hard on me”* (P.7). Others believed that, under favorable conditions such as adequate nutrition, economic stability, and strong spousal support, frequent childbearing would not necessarily harm women’s health:

*“My sister-in-law has ten children and is expecting the eleventh. She has no economic problems*,* and her health is fine because her husband supports her.”* (P.2).

While older participants often emphasized their resilience toward motherhood — *“Motherhood wasn’t hard for me. I wasn’t healthy*,* had no help*,* but still raised seven kids”* (P.5) — and many others, across ages, described motherhood as naturally challenging and an unavoidable part of a woman’s life — *“It is the path every woman must walk”* (P.13) — In contrast, a smaller group of younger women voiced concerns about personal health and body image: *“Childbirth was very hard for me*,* two kids would be enough*,* but because of my husband*,* I’m forced to have three more”* (P.16); *“Having many children ruins your body*,* you gain weight”* (P.8).

#### Birth timing and spacing preferences

A preference for spacing pregnancies by two to three years was common among participants, including those who were not using contraception. As one woman explained, *“Three years is right for a mom’s body to fully recover and get its strength back before having another baby”* (P.15). Post-cesarean spacing was also mentioned: *“Because I had a C-section*,* there should be a three-year gap between pregnancies”* (P.2). Some women, however, preferred longer intervals of four to five years or more, allowing the first child to grow more independent before the next:

*“If it were up to me*,* I’d want my child to grow up first — to get out of diapers*,* handle themselves a bit*,* and understand right from wrong — and only then think about having another baby.”* (P.20).

Age-related considerations also shaped birth timing. While many participants viewed early marriage (~ 20 years) and immediate pregnancy as optimal, a few expressed flexibility. Among those favoring early pregnancies, several perceived later childbirths as riskier:

*“From what we’ve seen*,* if a girl gets married and has a baby when she’s really young*,* the delivery goes much faster. But if she marries later*,* there can be problems. Like*,* my cousin married at 29 and had her first baby at 31*,* and her uterus ruptured.”* (P.2).

To reach their desired number of children while avoiding later-age pregnancies, some preferred shorter gaps: *“The only reason I want another baby sooner is my age. I’m 31 now*,* and if I wait*,* I’ll be 35*,* and it’ll get harder”* (P.2).

#### Health concerns regarding modern contraceptives

Among the 23 women interviewed, 12 were using modern contraceptives, five relied on withdrawal, and six had no intention of preventing pregnancy (two of whom were breastfeeding unintentionally as birth control).

Concerns about side effects — such as heavy bleeding, infections, physical weakness, and mental health problems — were the most frequent reasons for avoiding or discontinuing modern methods: “*I can’t take pills because women who have used them say they affect the nerves.*” (P.23) In addition, discontinuation due to adverse effects emerged as a frequent challenge among contraceptive users. Some participants attributed symptoms not well-documented in the literature to contraceptives: “*I had become irritable and experienced heart palpitations from taking the pills*,* so I stopped.*” (P.5).

Participants also repeatedly reported unintended pregnancies after contraceptive failure or discontinuation due to side effects:

“*After my daughter*,* who is now 11*,* turned one*,* I got an IUD. I kept it for two years*,* but I experienced heavy bleeding*,* so I had it removed. Then*,* my youngest daughter was born*.” (P.9).

Notably, male-oriented contraceptive methods were relatively common: six participants reported condom use, and four relied on withdrawal. These preferences were often linked to mistrust of hormonal or insertable methods:

“*I don’t take pills*,* and my husband takes precautions (using the withdrawal method). He is considerate of my health and tells me*,* ‘I don’t want you to get sick again; I’m tired of taking you to the hospital.’*” (P.3).

Some women experienced recurrent failure with these methods: “*I have been using condoms for 15 or 16 years*,* but I still got pregnant with my two daughters while using them*” (P.15). Persistent failures also led a few women to permanent solutions:

“*I experienced heavy bleeding and headaches with the IUD. I had it for three years*,* then had it removed and switched to the implant. I kept the implant for two or three years*,* but I still had headaches. After removing the implant*,* we used condoms*,* but I still got pregnant. After my child was born*,* I went to a private hospital and had surgery*.” (P.11).

### Emotional and psychological motivations of childbearing

#### Joy and companionship

Children were mainly described as a primary source of emotional warmth and connection:

*“When children are around*,* you never feel a sense of lack. They become your companions*,* just like your parents and friends. A child can never truly distance themselves from their parents. Children are the greatest blessing in a person’s life.”* (P.13).

Among women with fewer social outlets, the daily joy and companionship derived from children appeared particularly salient. As one illiterate housewife explained:

*“If I have a small child*,* my time is spent with fun. I’m alone in the house now. When I see babies anywhere*,* in surroundings that have a baby*,* it makes me envious. I would like to have another baby.”* (P.10).

While the emotional pleasure from children was acknowledged across backgrounds, some balanced it against the responsibilities of child-rearing. As a woman with a high school education said:

*“A laughing child is a joy*,* of course*,* but their upbringing is just as important. You can’t just let them play and break things*,* thinking it’s all part of having fun. You need to guide them*,* make them aware of what is right and wrong*,* and teach them from an early age. I don’t want a child just for entertainment.”* (P.12).

#### Fulfillment and marital stability

Motherhood was also viewed as central to a woman’s identity and a source of life purpose. One participant explained, “*If I had no children*,* I would feel like I was nothing at all*” (P.1), while another reflected more broadly, “*Even if you have everything else*,* without a child*,* you would not be truly happy*” (P.20).

Some participants emphasized that children were not only a source of personal fulfillment but also essential to the marital bond:

*“If I had no children*,* I would be deeply heartbroken. Children are the beauty and essence of a marriage. Without them*,* marriage has no meaning.”* (P.13).

The presence of children was often linked to stronger marital commitment, as one participant articulated: *“When a child is born*,* things get better. The husband becomes attached to the child*,* and the wife does too”* (P.16).

Some accounts suggested that ongoing restrictions on women’s education and employment were influencing reproductive choices in the community. One participant explained that, after their daughters’ education was interrupted, they were led to early engagement or marriage:

*“My daughter finished grade 12 and studied economics*,* but never received her certificate. When the Taliban came*,* she could no longer get it or work. Now she is engaged.” (P.4)*.

## Discussion

In this qualitative study of married women in Kabul, fertility attitudes were expressed across several dimensions, including socio-cultural expectations, religious and moral beliefs, economic considerations, health-related views, and emotional meanings attached to children. Socio-cultural norms strongly favored early childbearing and larger families and placed a high value on having sons, reinforced by husbands and mothers-in-law, while some women—often among the younger or more educated participants—described negotiating for fewer children or longer birth intervals within these expectations. Religious and moral interpretations were central to how women talked about contraception: many repeated messages that preventing pregnancy could be sinful or interfere with divine will, while some narratives also tried to reconcile faith with birth spacing by framing contraception as a way to protect maternal and child well-being or to meet parental responsibilities. Economic accounts reflected tension between worries about limited household resources and rising living costs, while children—especially sons—were simultaneously described as long-term providers of security and support for their parents. Health-related narratives highlighted the physical strain and heavy workload of childbearing and childcare, especially the burden of closely spaced, high-parity pregnancies; yet, some women portrayed these demands as an expected part of women’s lives or as less problematic when strong economic and familial support was available. At the same time, participants expressed concerns about the side effects of modern contraceptive methods, and their narratives included misconceptions about these methods as well as repeated method failures. Despite these burdens and concerns, children were described as a major source of companionship, identity, and marital stability, while a number of women also voiced unease about having more children than they felt able to raise “well.” Across these dimensions, women’s preferences were articulated within household hierarchies, with some accounts pointing to emerging orientations toward smaller families, longer birth intervals, and greater attention to children’s well-being. In the following sections, we interpret and discuss these dimensions in relation to previous Afghan and international research and the current socio-political context.

### Socio-cultural norms, son preference, and women’s agency

Our findings on socio-cultural norms and expectations reflected enduring patterns supporting large families, expressed through both collective comparisons and socio-familial expectations. The reported desired number of children among women implies that many still internalize prevailing social norms favoring large families. Meanwhile, social and familial pressures, especially from husbands and mothers-in-law, often pushed women further toward continued childbearing, which, at a broader level, helps sustain higher fertility patterns. Similarly, recent estimates place Afghanistan’s total fertility rate at 5.3 [31], and prior research shows that men tend to desire 6–7 children, compared with 3–4 among women [34], findings that are broadly consistent with ours. Previous studies have also identified husbands and in-laws—particularly mothers-in-law—as influential actors in fertility decision-making, favoring larger families, and as barriers to contraceptive use [11, 12, 33, 34]. Overall, high fertility appears to remain a dominant cultural pattern, sustained through both internalized normative perceptions and external enforcement mechanisms within families and communities.

Earlier Afghan research has also found lower contraceptive use among married Afghan women under 20, linking it to newly married status and low intention to avoid pregnancy [26]. Some studies also linked reluctance to use contraception to fears of lifelong infertility [11, 33, 34]. Taken together with our findings on expectations for very early first pregnancy, these familial expectations and fears of infertility seem to influence women’s early reproductive choices, potentially limiting contraceptive uptake and sustaining high fertility in this context.

These accounts illustrate how reproductive expectations intersect with gender norms to reinforce stigma and emotional strain, linking women’s social worth to their fertility outcomes—particularly to bearing sons, who embody both symbolic value and the perceived assurance of family continuity and heritage. Prior studies similarly noted that Afghan culture often associates a woman’s social value with her reproductive success—especially the birth of sons [12, 33].

Participants’ accounts suggest that while the cultural value of large families remains strong, some couples are beginning to express a preference for smaller families and improved living conditions, indicating that these values are gradually emerging in specific contexts.

In contrast to earlier studies that identified husbands and mothers-in-law as primary decision-makers [11, 34], our findings—while acknowledging the continued influence of husbands and in-laws—indicate conditional and relational forms of agency, in which women participate in or negotiate fertility decisions with spousal approval. In most cases, such familial influence was indirect, expressed through emotional expectations, persuasion, and other forms rather than overt restriction or control. Overall, the narratives suggest an emerging pattern of reproductive agency characterized by increasingly communicative and negotiated decision-making within marital relationships. These patterns, shaped by women’s access to information, their ability to engage in discussion with spouses, and the boundaries set by family expectations, are consistent with broader understandings of empowerment that view agency as context-dependent and influenced by available resources [36, 37]. In line with these perspectives, Afghan women with higher education, income, and access to family planning information and services are better positioned to exercise reproductive agency, and expanding these supports could further advance rights-based family planning initiatives, as highlighted in Afghan literature [2, 26, 33]. Moreover, evidence from Afghanistan and other low-resource settings recommends engaging men to support women’s reproductive rights and fostering communicative couple relationships to enhance women’s agency in fertility decisions [2, 11, 13, 33, 38–40].

Despite evolving fertility attitudes, shifting cultural values, and greater female participation in fertility decision-making, participants’ narratives consistently reveal that son preference remains deeply embedded and strongly influential across backgrounds. This enduring preference, reinforced primarily by husbands and in-laws, often led women to have more children than they originally intended.

Prior Afghan studies have likewise documented this phenomenon as a prominent feature of fertility attitudes in Afghanistan [2, 12, 33, 34]; one qualitative study noted that while many families consider four to five children acceptable, they may have up to ten in pursuit of sons [12].

The insights in this category illustrate the persistence of deeply rooted pronatalist and patrilineal norms, particularly son preference, which continue to influence women’s reproductive decisions despite emerging shifts in fertility attitudes. Expectations for early and repeated childbearing, often reinforced by family elders, frequently override women’s own preferences. At the same time, women’s narratives reflect conditional forms of agency as they negotiate their preferences within the boundaries set by family expectations.

### Religious interpretations and ethical reasoning about fertility

Participants’ views about the sinfulness of deliberately preventing pregnancy have also been documented in earlier Afghan studies [11, 12, 34, 41], and are consistent with the evidence from Muslim-majority contexts where fertility is closely connected to religious values [42]. The parallel perception that contraception is considered acceptable within Islamic teachings, while abortion is not, is also noted in previous research. These studies highlight that although some religious scholars oppose family planning, others support it within an Islamic context, but strongly reject elective abortion [11, 33].

Earlier studies have similarly noted that some families regarded children as divine gifts that should not be refused [11, 12, 33], and that certain religious scholars frame population growth among Muslims as a religious duty [11]. Within this context, the 2019 UNFPA Afghanistan report remarked that engaging supportive religious leaders can help increase acceptance of family planning [43].

These perspectives offer more pragmatic, responsibility-based interpretations of religious morality, representing viewpoints not extensively described in previous Afghan qualitative research, which predominantly reported traditional fertility norms. Women’s own interpretations and moral reflections shed light on how many reconcile faith with the economic and physical demands of high fertility. Similar patterns have been noted in studies showing that ethical and health-focused framing of family planning can improve its acceptance in Islamic contexts [33, 44, 45]. In Afghanistan, experience has shown that framing family planning in ways that emphasize maternal and child health for birth spacing is more widely accepted than advocating for smaller families [33]. Building on this, culturally sensitive approaches grounded in health and ethical considerations may further enhance the effectiveness of family planning strategies.

### Economic burdens of child-rearing and the long-term functional value of children

Drawing on participants’ accounts, many women in this study tended to adjust their desired family size to available resources, a pattern that *echoes* economic perspectives such as Becker’s Microeconomic Theory of Fertility [46]. However, earlier Afghan studies only briefly touched upon the impact of financial constraints on fertility decisions [11, 34]. During the two decades preceding the 2021 political transition, Afghanistan experienced notable economic and social transformations. These changes included expanded access to education, rapid urbanization, and greater media exposure [47–49], which may help contextualize the attitudes toward family size reflected in our data. These observations also align with broader demographic perspectives, such as the Demographic Transition Theory [50], which suggests that as societies undergo socioeconomic development, fertility preferences shift from larger to smaller families, driven by greater attention to child quality rather than quantity.

Additionally, following the sharp decline in international aid to the country, recent economic hardship has further strained household budgets. Combined with a shift in childbearing values, this situation may have led some families to reassess the costs and responsibilities of having children. Further quantitative research is needed to measure the prevalence of these attitudes and the extent to which they are put into practice.

For some rural participants, however, traditional faith-based attitudes toward fertility appeared more salient than economic considerations, a pattern that has also been noted in other Muslim-majority societies [51]. This suggests that, for some families, deeply rooted religious values may outweigh financial concerns when making fertility decisions.

This belief converges with the ‘old-age security’ hypothesis [52, 53], which explains higher fertility in countries with limited formal pension systems. Previous Afghan studies also document children’s economic and support roles—especially sons—as key drivers of larger family preferences [11, 12, 33, 34]. In this study, participants mainly referred to the traditional expectation that sons provide financial security and long-term care for their families.

These perspectives illustrate how some women are beginning to reassess intergenerational support and align with patterns documented in other studies, which show that urbanization, economic hardship, and women’s increasing participation in income-generating activities can reshape family dynamics and influence gender preferences [36, 54–56]. Nevertheless, such perspectives remain largely underexplored in Afghan research.

Participants’ narratives also pointed to an emerging orientation toward valuing children’s well-being and personal development over their economic utility. Similar child-centered values have been documented only rarely in Afghan research [34].

### Health, contraceptive concerns, and women’s well-being

The women’s narratives reveal the substantial physical strain and heavy workload that they face from repeated pregnancies, childcare, and household duties, which—within this cultural context—often compromise their own health. While occasional family or spousal support offered some relief, social expectations and familial pressures frequently compelled continued childbearing despite fatigue or illness.

Many participants viewed the hardships of motherhood as an inevitable part of women’s lives, reflecting cultural adaptation and internalized social expectations [57, 58]. However, most emphasized that these burdens were further intensified by additional physical, emotional, and economic pressures beyond motherhood itself. Some women believed that under ideal living conditions, frequent childbearing would be harmless, but clinical evidence shows that high-parity pregnancies inherently increase health risks [59].

In Afghanistan’s low-resource context, prior studies consistently show that women’s well-being is often subordinated to family expectations and overlooked within dominant social norms [12, 42, 60, 61]. These findings suggest the importance of integrated, context-sensitive family planning strategies aligned with UNFPA’s global framework [62]. Such approaches may need to focus on challenging restrictive norms, addressing misconceptions, and supporting women to make more informed and health-protective reproductive decisions within the constraints they navigate.

While our findings reveal more diverse perspectives, previous Afghan studies reported preferred intervals of 2 to 5 years, mainly for maternal and child health; however, despite broad awareness of health benefits, shorter intervals remain common due to social pressures and structural barriers [11].

Women’s accounts also showed that concerns about side effects—whether based on experience, misunderstanding, or community narratives—significantly shape contraceptive behavior and contribute to unmet need. Existing Afghan literature has highlighted prevailing concerns, complaints, and misconceptions regarding the side effects of modern contraceptives among Afghan women [11, 13, 34]. A notable mistrust of hormonal and device-based methods also echoes prior findings on the cultural preference for natural options like withdrawal [2, 34]. Addressing these barriers through comprehensive family planning counseling, provision of safer and more reliable contraceptives, adequate education, consistent follow-up care, and culturally sensitive community engagement strategies may enhance acceptance and continuity of contraceptive use, ultimately supporting more informed and health-protective reproductive choices.

### Emotional meanings of childbearing and gendered identities

Our findings, alongside other Afghan studies, also indicate that many families associate larger households with greater happiness [11]. These emotional motivations reflect a broader pattern typical of low-resource settings, where limited opportunities for leisure or self-fulfillment make children central sources of emotional value and satisfaction [53].

Prior research indicates that Afghan women mainly perceive motherhood as central to feminine identity and social value, reinforced by familial expectations [63]. In contrast, in more modernized societies, emotional satisfaction more often comes from career, autonomy, and personal achievements rather than motherhood alone [64, 65], which may support freer fertility choices.

However, recent restrictions on women’s education and employment further limit success pathways for girls and women. Participants’ narratives suggested that, in the absence of alternative roles or opportunities, marriage can become the natural next step for young women, and, combined with the social expectations about early pregnancy just after marriage, it can contribute to starting childbearing at earlier ages.

Recent and ongoing community- and health-based initiatives—such as UNDP’s Enhancing Gender Equality and Women’s Empowerment (EGEMA), CARE International’s Livelihoods and Integrated Health Strengthening Project, and national NGO-led efforts like the Empowerment Center for Women (ECW)—have sought to strengthen women’s skills, economic participation, and reproductive health awareness through local outreach. While programs like EGEMA primarily focus on women’s economic and social empowerment, projects led by CARE and ECW directly integrate education on maternal and reproductive health, including family planning and birth spacing, into their community-based activities [23–25]. Together, these efforts can enhance women’s confidence, self-efficacy, and awareness, contributing—both directly and indirectly—to more informed fertility decisions and improved reproductive well-being under current constraints [26, 33]. However, achieving sustainable improvements in women’s reproductive agency ultimately depends on restoring equitable access to education and employment opportunities.

### Strengths and limitations

This study’s main strength lies in amplifying women’s voices from a highly restricted and vulnerable context through a rights-based lens aligned with global frameworks of reproductive autonomy. Centering women’s narratives in this study provides nuanced insights into their attitudes, including emerging preferences for smaller families, evolving gender roles, and greater attention to child well-being.

As with most qualitative research, the findings are context-specific rather than intended for broad transferability, yet they offer in-depth perspectives from a purposively selected, diverse group of married women in Kabul. However, full participant characteristics are provided to help readers assess the relevance of these findings to other settings. Although the single-province setting and the recruitment of married women—mainly among hospital companions and through personal academic, clinical, and community networks—limit the applicability of the findings to women with similar profiles, maximum variation sampling helped enhance the credibility and richness of the data, and this sampling context should be considered when interpreting the views presented in this study. Although maximum-variation sampling was applied, parity was not included as a criterion to ensure variation; consequently, all participants happened to be mothers, which should also be taken into account when interpreting the findings. Additionally, pregnant women were excluded to explore fertility attitudes unaffected by current pregnancies, and subgroup differences were not systematically analyzed in this study, although some variations were observed, suggesting potential directions for future research. The descriptive numerical information reported is only to profile the sample, and findings should be interpreted within the demographic and contextual boundaries of this study.

The study also encountered several practical constraints, including time-consuming administrative permissions, challenges securing private interview spaces, and some participants’ reluctance to audio-record, which required detailed manual interview notes. Cultural reservations about participation further required additional efforts to build trust and reassure participants about confidentiality, while early communication about recording options helped manage expectations and reduce potential dropout. Obtaining written informed consent was occasionally difficult due to varying levels of literacy and hesitancy to sign formal documents; in these cases, the study procedures and consent information were explained orally, and participants’ agreement was documented either through an audio-recorded statement or a witness signature indicating that oral consent had been provided.

## Conclusion

Since 2021, the widespread restrictions on Afghan women’s education, employment, mobility, and access to care have reshaped daily life and intensified existing reproductive vulnerabilities. In a context already marked by persistently high fertility and one of the world’s highest maternal mortality ratios, understanding how women think about and navigate childbearing is essential for protecting existing gains and anticipating future needs.

Women’s narratives in Kabul illustrate how deeply rooted socio-cultural and religious expectations continue to guide fertility decisions, while many also raised concerns about their own health, their children’s well-being, the demands of large families, and the need for longer intervals between pregnancies. Women who voiced these concerns often relied on negotiation within their households, and younger, educated, and employed participants tended to achieve better outcomes.

Women’s accounts point to three consistent themes: caring for their own health, wanting to provide well for their children, and using subtle forms of negotiation to express their preferences. Taken together, these experiences reflect orientations consistent with more responsibility-based and health-oriented approaches to childbearing, even though such preferences still unfold within strong pronatalist expectations that can limit how fully women act on them.

Current restrictions on education, employment, and mobility pose significant risks to these orientations. Over the long term, the loss of schooling and work not only reduces women’s influence in household decision-making but also narrows alternative identity and achievement pathways beyond motherhood—conditions that may reinforce pronatalist expectations. In the short term, reduced mobility and limited access to reliable information further restrict opportunities to obtain reproductive guidance, even though many health facilities continue to operate and no formal ban on reproductive services has been issued.

### Implications for practice

Within the current constraints, policy responses should focus on what remains realistically feasible. Discreet and culturally acceptable ways of providing accurate reproductive and birth-spacing information through trusted providers, alongside efforts to support respectful communication between spouses, can help preserve small but meaningful gains and reduce maternal and child health vulnerabilities. Given the growing emphasis on children’s well-being and manageable caregiving, approaches framed around child health and family stability may resonate more strongly than messages centered on limiting fertility. While broader social and cultural barriers are difficult to engage with directly in the current context, gentle, low-profile interactions through existing health contacts may offer opportunities for incremental improvement.

Recognizing the health-oriented and responsibility-driven attitudes reflected in women’s accounts can guide context-sensitive strategies to safeguard minimal progress and mitigate preventable risks. Yet these emerging shifts remain fragile, and their long-term impact is uncertain unless broader constraints on women’s lives are eased.

## Data Availability

The datasets generated and analyzed during the current study are not publicly available due to confidentiality concerns but are available from the corresponding author on reasonable request.
